# Inclusive musical environments in educational contexts: a conceptual framework for equity, participation, and student wellbeing

**DOI:** 10.3389/fpsyg.2026.1923071

**Published:** 2026-10-12

**Authors:** Lorena Alleres-Otero, Cristina Honrubia-Montesinos, Luisa Losada-Puente, Pedro Gil-Madrona

**Affiliations:** 1 University of A Coruña, A Coruña, Spain; 2 Universidad Camilo Jose Cela, Villafranca del Castillo, Spain; 3 Facultad de Educacion, Universidad de Castilla-La Mancha, Ciudad Real, Spain; 4 Universidad de Castilla-La Mancha, Ciudad Real, Spain

**Keywords:** educational equity, gender equity, inclusive education, music education, musical participation, sense of belonging, student wellbeing

## Abstract

This review develops a framework for examining the conditions under which musical environments may support equity, participation, and situated student wellbeing in educational contexts. To this end, it brings together literature from music education, educational psychology, inclusive education, music therapy, and critical studies of gender, disability, and intersectionality. Recent research has linked music education with self-esteem, belonging, social–emotional learning, emergent literacy, movement, and inclusion; however, there is extensive heterogeneity in designs, populations, measures, and contexts. Accordingly, the article does not begin from the premise that music is inclusive, but from the need to identify the pedagogical and relational conditions that make equitable musical participation possible. Peer-reviewed studies published between 2018 and 2026 were analyzed by distinguishing three evidence domains: studies and reviews with direct implications for school contexts; broader educational scholarship from higher education, ensembles, and music clubs; and complementary therapeutic evidence. In light of this body of research, musical inclusion can be organized around three mechanisms of preventive equity: multimodal pedagogical access and situated representation; recognized participation and voice; and expressive safety, belonging, and situated wellbeing. The article argues that music may support equity when its pedagogical design broadens the legitimate ways of learning, participating, and contributing within a shared practice. By doing so, the article shifts the discussion from the general benefits of music toward the conditions that make a musical experience inclusive, at a time when recent research is increasingly examining musical participation in relation to wellbeing, belonging, identity, agency, assessment, and institutional conditions.

## Introduction

Recent literature does not allow music to be assumed as an inclusive practice merely because it is present in an educational setting. Reviews on music education and psychoeducational dimensions such as wellbeing, self-esteem, social–emotional learning, emergent literacy, and inclusion describe positive associations, while also pointing to heterogeneity in designs, measures, populations, contexts, and interventions (Blasco-Magraner et al., 2021; Eccles et al., 2021; Goopy and MacArthur, 2025; Jiang and Tong, 2025; Mommo et al., 2025; Váradi, 2022). This heterogeneity matters because the outcomes associated with musical practices cannot be understood independently of the pedagogical, social, and relational conditions in which those practices are organized. The same musical activity may broaden participation for some students while leaving others in peripheral roles if access, recognition, expressive exposure, assessment, and support are not carefully structured. For this reason, the conceptual problem addressed by this framework is not whether music has educational potential, but which pedagogical and relational conditions allow a musical practice to open or restrict possibilities for participation, identity recognition, and student wellbeing.

In music education, inclusion has been defined and operationalized in different ways according to national policies, curricular traditions, and institutional cultures. Chiba and Hebert (2025) showed that the meaning of inclusivity varies across educational systems and that standardized curricular formats can hinder the participation of diverse students. This finding suggests that inclusion cannot be examined only as formal access to the classroom, the instrument, or the repertoire. It must also be analyzed in relation to the curricular, pedagogical, and institutional formats that determine whether diverse students can participate meaningfully within musical practice.

The systematic review by Mommo et al. (2025) on music lessons with students with special educational needs showed that inclusion is often implemented as physical integration into the mainstream classroom. Effective participation, however, depends on concrete supports, peer interaction, professional collaboration, and methodological diversity. These findings shift the analysis from physical presence toward the quality of pedagogical support, opportunities for participation, and the recognition of different ways of learning and contributing musically.

The pedagogical function of music in educational contexts has been studied from different perspectives. Eccles et al. (2021) reviewed music interventions with children aged five to eight and found improvements in some aspects of phonological awareness and early literacy, although the number of eligible studies was small and methodological diversity was substantial. Del Barrio and Arús (2024) examined music and movement pedagogy in basic education and positioned the body as a pathway for musical representation, understanding, and experience. These syntheses support the claim that music can offer multimodal pathways for learning, provided that an automatic transfer of skills is not assumed.

Student wellbeing is incorporated in this analysis as a dimension dependent on the educational environment, not as an inherent effect of musical activity. Váradi (2022) linked music education with social–emotional learning, empathy, cooperation, and social relationships. Similarly, Jiang and Tong (2025) analyzed studies on self-esteem in music education contexts and observed a generally positive relationship, with limitations stemming from predominantly cross-sectional designs and uneven methodological quality. Goopy and MacArthur (2025) examined the relationship between music learning and wellbeing among school-aged students, describing individual, social, and educational outcomes. In this article, “school-aged students” refers primarily to children and adolescents enrolled in primary and secondary education. When broader studies include community-based music learning or participants close to the transition into adulthood, their findings are interpreted cautiously according to the body of literature from which they come. Emotional wellbeing is therefore influenced by pedagogical and relational conditions: support, recognition, expressive safety, belonging, and sustained participation.

To refine the understanding of these relational mechanisms, literature from ensembles, university music clubs, and music therapy was used in a complementary way. Ryan et al. (2025) identified belonging, leadership, atmosphere, identity, and wellbeing as recurrent themes in relationships within ensembles. Guo et al. (2026) observed increases in belonging, identity development, agency, perceived recognition, and expressive safety after participation in university music clubs, although their results should be read cautiously because of the exploratory design. These findings help specify relational dimensions that inform the analysis of educational environments: who can take initiative, who receives recognition, how leadership is distributed, and what relational consequences arise from musical exposure before others. In music therapy, recent work on queer and trans practice has emphasized the role of identity recognition, affirming language, relational safety, and awareness of power asymmetries in musical participation (Baines et al., 2019; Mulley et al., 2026; Ribeiro et al., 2025). These contributions are integrated into the framework to delimit relational conditions, such as recognition, trust, and language, that cut across different musical environments. Taken together, these bodies of literature point to three recurring analytical problems in musical inclusion: how students gain access to musical practice and encounter legitimate forms of representation; whether their participation becomes recognized contribution and voice; and how expressive exposure, error, belonging, and wellbeing are relationally regulated. These problems did not emerge as isolated themes but were refined through an iterative reading of the literature in relation to the aims of the article. They provide the basis for the three mechanisms developed in the framework.

This article develops a conceptual framework for examining inclusive musical environments in educational contexts. The expression “educational contexts” refers here to compulsory schooling, formal music education, community-based educational practices, and higher education. Therapeutic literature is included only when it provides conceptual tools for understanding recognition, relational safety, power, and participation, without treating these contexts as institutionally or pedagogically equivalent.

The article is guided by the following question: under what conditions can musical practices transform formal access into meaningful, recognized, and equitable participation? This question responds to a tension in the literature: while music education has often been associated with wellbeing, belonging, social–emotional learning, and inclusion, these outcomes cannot be assumed to emerge from musical participation itself. They depend on how access, recognition, voice, evaluation, expressive exposure, and institutional responsibility are organized within specific educational environments.

Preventive equity is understood here as an analytical orientation that examines the pedagogical, relational, and institutional conditions that may generate exclusion before such exclusion becomes visible as low participation, silence, loss of confidence, peripheral roles, or withdrawal. What is being prevented is not difference, difficulty, or diversity itself, but the consolidation of avoidable barriers that progressively restrict students’ possibilities for meaningful musical participation. In this sense, preventive equity does not refer to a tested intervention model, but to a conceptual lens for identifying conditions that can either expand or restrict students’ possibilities for meaningful participation in musical practices.

Conceptually, preventive equity is not proposed as a wholly new educational theory or as an empirically validated intervention model. Rather, it is used as an integrative lens that brings together principles already present in Universal Design for Learning, inclusive pedagogy, social justice education, and anticipatory approaches to educational design. It shares with Universal Design for Learning the concern with designing flexible routes for access, engagement, representation, action, and expression (CAST, 2024; Meyer et al., 2014); with inclusive pedagogy, the rejection of deficit-based responses to learner diversity (Florian and Black-Hawkins, 2011); and with social justice-oriented music education, attention to power, recognition, bias, and unequal participation (Campbell, 2020; Harkopf and Bylica, 2023). Its specific use in this article lies in applying these concerns to musical environments, where repertoire, bodily and vocal exposure, assessment, leadership, musical identity, and material access shape who can enter, contribute, remain, and progress.

The general aim of the article is to examine recent scientific literature on music education and related fields in order to identify and systematize the mechanisms that configure conditions of preventive equity in musical participation. To address this aim, the review pursues four objectives: to examine how access and participation are constructed in musical practice by identifying the pedagogical and social criteria that legitimize different forms of contribution; to clarify the role of musical mediation and multimodal pedagogical design in relation to learning, participation, and inclusion; to identify the conditions that shape recognized participation, voice, and expressive safety, with attention to relational, evaluative, and identity-related factors; and to define the role of teacher facilitation and institutional responsibility in sustaining equitable musical practices.

The three mechanisms were not established *a priori* as fixed categories, nor do they follow from the objectives alone. They were developed through an iterative process in which the objectives guided the initial reading and the comparative synthesis of the literature progressively refined the analytical categories. Through this process, recurring concerns related to access and legitimacy, recognized contribution and voice, and the relational regulation of expressive exposure, belonging, and wellbeing were grouped into three mechanisms of preventive equity: multimodal pedagogical access and situated representation; recognized participation and voice; and expressive safety, belonging, and situated wellbeing. These mechanisms are therefore proposed as analytically distinct but interdependent conceptual dimensions derived from the integrative synthesis, rather than as empirically validated categories.

## Methodology

This study is based on an integrative narrative review with a conceptual orientation. This approach was considered appropriate because the manuscript addresses a broad, heterogeneous, and interdisciplinary field in which empirical studies, systematic and scoping reviews, conceptual analyses, and critical scholarship converge. Unlike a systematic review aimed at estimating effects or exhaustively mapping a closed body of evidence, an integrative narrative review allows evidence from different but related fields to be interpreted, compared, and organized in order to develop a theoretically grounded conceptual synthesis (Baumeister and Leary, 1997; Dixon-Woods et al., 2006; Grant and Booth, 2009; Greenhalgh et al., 2018).

The review focused on peer-reviewed literature published mainly between 2018 and 2026. This period was selected in order to capture recent debates on inclusive music education, educational wellbeing, social–emotional learning, intercultural competence, gender, disability, intersectionality, and music-based practice. Earlier sources were included only when they provided methodological justification for the review approach or when they were necessary to support the conceptual framing.

Searches were conducted in Web of Science and Scopus on 6 May 2026 and updated on 11 September 2026. Web of Science and Scopus were selected as the principal databases because, together, they provide broad coverage of peer-reviewed literature across education, psychology, social sciences, arts and humanities, and health-related fields, which was appropriate for the interdisciplinary scope of the review. Records retrieved from both databases were exported to a reference-management file and compared before screening. Duplicate records were identified by DOI, title, authorship, and publication year; when the same source appeared in both databases, one record was retained and the duplicate was removed before title and abstract screening. The search strategy combined terms from five thematic blocks: music education/music learning/music therapy; inclusion/equity/belonging/participation/agency; wellbeing/self-esteem/social–emotional learning; special educational needs/disability/intercultural competence; and gender/queer/intersectionality. The following boolean combinations guided the searches:

(“music education” OR “music learning” OR “music therapy”) AND (inclusion OR equity OR belonging OR participation OR agency)

(“music education” OR “music learning”) AND (wellbeing OR “self-esteem” OR “social–emotional learning”)

(“music education”) AND (“special educational needs” OR disability OR “intercultural competence”)

(“music education” OR “music therapy”) AND (gender OR queer OR intersectionality)

These searches were complemented by manual screening of references cited in selected reviews and articles. This complementary search was used to identify conceptual or field-specific work that directly informed the framework but might not have appeared consistently across database searches because of differences in terminology between music education, educational psychology, inclusion studies, and music therapy.

Studies were included when they met at least one of the following criteria: they addressed music education or music-based practices in educational, community, ensemble, higher education, or therapeutic contexts; they examined inclusion, participation, belonging, wellbeing, agency, gender, disability, intercultural competence, recognition, or expressive safety; or they provided conceptual tools for examining barriers and enabling conditions in musical participation. Priority was given to systematic reviews, scoping reviews, recent empirical studies, conceptual articles, and critical scholarship with an explicit connection to the aims of the manuscript.

Studies were excluded when they did not focus on music or music-based practice, did not address educational, relational, or participatory dimensions, were not peer-reviewed, or did not provide transferable insights for examining inclusive musical environments. Because the aim was conceptual synthesis rather than effect estimation, studies were not excluded on the basis of methodological design alone. Instead, their role within the review was differentiated according to the type and analytical function of evidence they provided.

The final conceptual corpus included 28 music-related substantive academic sources, three additional theoretical or field-guideline sources used to position preventive equity in relation to Universal Design for Learning and inclusive pedagogy (CAST, 2024; Florian and Black-Hawkins, 2011; Meyer et al., 2014), and four methodological references used to justify the review design. The substantive corpus included systematic reviews, scoping reviews, empirical studies, conceptual articles, critical scholarship, and scholarly book chapters or edited volumes when they directly addressed the aims of the manuscript. The final synthesis was limited to sources available in English. Because the review was integrative and conceptual rather than systematic, the searches were not used to produce a PRISMA-style exhaustive count of records. Instead, sources were retained when they contributed directly to at least one of the analytical dimensions of the framework: access and legitimacy, participation and voice, expressive safety and wellbeing, or teacher and institutional mediation.

Following these criteria, the selection process proceeded in three stages. First, titles and abstracts were screened to identify sources addressing music, inclusion, participation, wellbeing, identity, or equity-related dimensions. Second, full texts were reviewed when the abstract indicated a possible contribution to the conceptual aims of the manuscript. Third, selected sources were assigned to one of three evidence domains according to three criteria: the educational context examined, the population addressed, and the analytical function of the source within the framework. Sources involving school-aged students or direct school-level pedagogical implications were classified as direct educational evidence. Sources from higher education, ensembles, music clubs, community music, or policy-oriented music education were classified as expanded educational evidence when they helped specify belonging, voice, leadership, agency, assessment, or institutional culture. Music therapy sources were classified as complementary therapeutic evidence when they contributed concepts related to recognition, language, relational safety, identity, or power, without being treated as direct evidence for school-level educational claims.

Data extraction focused on six dimensions: study context, population, theoretical constructs, forms of musical participation, barriers to inclusion, and implications for pedagogical or institutional design. These elements were recorded in an analytical matrix and compared across the three evidence domains. The purpose of this comparison was not to aggregate findings, but to identify recurring conceptual patterns and tensions across fields.

The analysis combined deductive and emergent coding. Deductive categories were derived from the aims of the article and included access, participation, voice, wellbeing, recognition, support, assessment, and institutional responsibility. During comparative reading, these categories were refined through emergent codes, including expressive exposure, situated representation, peripheral participation, musical confidence, relational safety, and legitimacy of contribution. Through this iterative process, related codes were grouped into three organizing mechanisms: multimodal pedagogical access and situated representation; recognized participation and voice; and expressive safety, belonging, and situated wellbeing.

The initial screening, extraction, and coding were conducted by the first author, who was responsible for the formal analysis and methodological design of the review. The resulting categories and their organization into mechanisms were subsequently discussed by the author team during the revision of the manuscript. Analytical decisions were guided by the recurrence of concepts across sources, their connection to the guiding question, and their capacity to explain conditions that may enable or restrict equitable musical participation. Teacher facilitation and institutional responsibility were coded separately at first, but during the synthesis they appeared across the categories associated with the three mechanisms rather than as an independent dimension of student participation. For this reason, they were conceptualized as transversal enabling conditions.

The three mechanisms are therefore not presented as empirically tested dimensions of a validated model. They are conceptual propositions generated through an integrative synthesis of the reviewed literature. Their purpose is to provide analytical criteria for examining the conditions under which musical environments may support or restrict equitable participation.

To make the evidentiary function of each body of literature explicit, Table 1 presents representative sources from the conceptual corpus, organized according to their proximity to school-based educational contexts and their analytical role in the construction of the framework.

**Table 1:** Representative sources by evidence domain and analytical function within the framework.

Evidence domain	Sources	Analytical function
School education	Antunes et al. (2025), Eccles et al. (2021), Blasco-Magraner et al. (2021), Del Barrio and Arús (2024), Goopy and MacArthur (2025), Mommo et al. (2025), and Concina and Gesuato (2025).	To support claims about pedagogical support, emergent literacy, music-movement, school wellbeing, special educational needs, and gender stereotypes in school-age groups.
Formal, higher, and community music education	Chiba and Hebert (2025), Crawford (2020), Ryan et al. (2025), Guo et al. (2026), Zhu and Li (2026), Borgström Källén and Ferm Almqvist (2025), and Parker and MacLeod (2026).	To specify transferable mechanisms with caution: belonging, agency, recognition, evaluative bias, leadership, roles, repertoires, and institutional culture.
Music therapy and intersectional approaches	Baines et al. (2019), Biedka (2022), Gross (2023), Mulley et al. (2026), and Ribeiro et al. (2025).	To enrich the ethical and relational analysis: safety, language, identity, power, recognition, and affirmative practice.

This distinction was used to avoid treating all sources as equivalent. School-based studies provided the most direct educational evidence, while higher education, community, ensemble, and therapeutic literature were used more cautiously to refine relational, institutional, and identity-related mechanisms.

## Results

### Conceptual mechanisms of preventive equity in musical environments

The results are presented as a conceptual synthesis rather than as empirical findings in the conventional sense. The three mechanisms identified below synthesize patterns observed across the reviewed literature and translate them into analytical criteria for examining inclusive musical environments. They are analytically distinct because each addresses a different dimension of participation: access and legitimacy, recognition and voice, and expressive safety and wellbeing. At the same time, they are interdependent, since students’ participation depends on the combined organization of access, recognition, relational safety, and institutional support.

### From access to participation

In the comparative synthesis, studies on special educational needs, interculturality, gender, assessment, and multimodal learning were first coded around two related categories: access routes and criteria of legitimacy. Access routes referred to the ways through which students could enter and participate in musical practice; criteria of legitimacy referred to the repertoires, instruments, bodies, cultures, and forms of response recognized as musically valid. These categories provided the analytical transition from the distinction between access and participation to the first mechanism: multimodal pedagogical access and situated representation.

Once formal access and effective participation have been differentiated, the analysis shifts toward the criteria that make a musical contribution be considered valid. In music education, these criteria operate through the curriculum, repertoires, available instruments, accepted forms of response, distribution of roles, and assessment procedures.

When these criteria are fixed uniformly, diversity may be admitted only insofar as it fits the dominant format. Intercultural literature has shown that the inclusion of diverse repertoires is not enough if these repertoires are not treated as situated, active, and contextualized practices (Campbell, 2020; Crawford, 2020; Tomé-Fernández and Bravo-Yebra, 2025). The issue is therefore not to expand the repertoire superficially, but to review which cultures, forms of musicality, and modes of learning are granted pedagogical legitimacy.

Studies on gender and assessment show how this legitimation can be distributed unequally. Concina and Gesuato (2025) documented stereotyped beliefs about the suitability of musical instruments among students in primary and early secondary education. Borgström Källén and Ferm Almqvist (2025) showed that gender inequality in higher music education is maintained through positions of prestige and periphery. Parker and MacLeod (2026) found effects of race and interactions among race, gender, and instrument in musical performance evaluations conducted by music teachers. Read together, these works indicate that access to musical practice also depends on the criteria that organize which bodies, instruments, repertoires, and forms of response acquire value within the environment.

From this perspective, preventive equity does not mean adding support after exclusion has appeared. It means reviewing in advance the conditions that may produce peripheral participation, silencing, or loss of musical confidence. The question becomes how the environment is organized so that different ways of learning, sounding, moving, leading, and responding can acquire pedagogical and relational value within a shared practice.

### Music as pedagogical mediation in educational contexts

It is useful here to distinguish among education in and about music, education through music for broader educational outcomes, and therapeutic uses of music. Education in and about music refers to musical learning itself: listening, performing, composing, improvising, developing vocal or instrumental technique, engaging with repertoires, participating in ensembles, and building musical identity and progression in musicianship. Education through music refers to the use of musical practice to support broader educational outcomes, such as literacy, self-regulation, social–emotional learning, self-esteem, or wellbeing. Therapeutic uses of music involve different aims, settings, and professional relationships, and are used in this review only as complementary conceptual evidence. This distinction prevents musical practice from being treated as a homogeneous activity and clarifies that inclusion concerns both access to wider educational outcomes and access to musical learning itself.

Within this distinction, musical practice can organize learning through sound, movement, auditory memory, temporal coordination, emotion, and social relationship. This pedagogical function is especially clear in the literature on music-movement. Del Barrio and Arús (2024) reviewed research in basic education and showed that movement functions as a means of representing, understanding, and experiencing music. Juntunen and Sutela (2023) added evidence on music-movement interventions with vulnerable groups, with results linked to self-regulation, social interaction, engagement, agency, auditory attention, and inclusion.

The relationship with emergent literacy specifies this mediation in early school contexts. Eccles et al. (2021) reviewed studies on Orff, Kodály, Suzuki, and Dalcroze approaches with children aged five to eight and found improvements in aspects of phonological awareness and early literacy. For the present framework, the point is not that music replaces language teaching or produces automatic transfer. Its value lies in the fact that some musical practices work on fine listening, rhythmic patterning, sound segmentation, repetition, auditory memory, and temporal coordination when they are explicitly designed.

The socioemotional dimension has been addressed through the relationship between music education, emotional recognition, cooperation, and social relationships. Váradi (2022) reviewed connections between music education and social–emotional learning. Blasco-Magraner et al. (2021) synthesized studies on the educational use of music with children aged 3 to 12 and identified effects on emotional development, academic performance, and prosocial skills. Antunes et al. (2025), in a school-based social–emotional learning program, incorporated activities with music, rhythm, movement, dramatization, and emotional expression within a structured intervention. Their results showed improvements in executive functions according to caregiver ratings, but not according to teacher ratings. This discrepancy reinforces a methodological caution of the framework: the effects of musical, bodily, and arts-based practices depend on design, context, and assessment procedures.

These findings should therefore be interpreted cautiously. They do not demonstrate that music-movement, emergent literacy, executive-function, or socio-emotional outcomes amount to inclusion or preventive equity by themselves. Rather, they suggest that, under certain pedagogical designs, musical practices may mediate learning through auditory, bodily, rhythmic, emotional, and social pathways. Their relevance for the present framework lies in the conceptual link between mediation and access: when these pathways are intentionally organized as legitimate forms of participation, they may broaden the ways in which students enter, represent, and demonstrate learning. In this sense, music as pedagogical mediation may contribute to preventive equity only when it is connected to inclusive design, recognition, and adequate support, rather than treated as an automatically beneficial activity.

### Multimodal pedagogical access and situated representation

In the synthesis, studies on music-movement, emergent literacy, special educational needs, intercultural competence, and gender did not point only to methodological variety. They converged around a broader analytical issue: whether students have several legitimate ways of entering musical practice and whether those ways of participating are recognized as musically and pedagogically valid. On this basis, multimodal pedagogical access refers to the availability of several legitimate pathways into musical practice, including listening, singing, moving, playing, creating, reading, improvising, responding bodily, engaging in dialogue, or participating through peer support (Del Barrio and Arús, 2024; Eccles et al., 2021; Juntunen and Sutela, 2023; Mommo et al., 2025). In this framework, multimodality is therefore treated as a conceptual proposition derived from the synthesis: inclusive musical design may require more than one pathway for access, meaning making, and demonstration of understanding.

This perspective avoids reducing inclusion to subsequent adaptation. The problem does not arise only when a student needs an individual accommodation; it arises when the musical activity has been designed around a single legitimate pathway of participation, such as notation reading, immediate verbal response, conventional instrumental performance, or public exposure without graduated support. Multimodal access places the diversity of entry points within the initial design of musical practice.

Situated representation completes this mechanism. Intercultural literature indicates that repertoires and materials are not neutral: they communicate which cultures, histories, languages, and forms of musicality receive legitimacy in the classroom or program (Campbell, 2020; Crawford, 2020). Tomé-Fernández and Bravo-Yebra (2025) showed that music teachers in Spain value intercultural competence, but identify lack of training, curricular rigidity, and limited advice as barriers. Situated representation requires contextualizing repertoires and practices within active pedagogical experiences, rather than adding cultural examples in isolation.

Multimodal access and situated representation are grouped within the same mechanism because both concern the initial conditions of legitimacy in musical participation. The first refers to how students can enter the practice; the second refers to which repertoires, bodies, cultures, histories, and forms of musicality are recognized as valid once participation begins. Together, they define whether access is merely available or whether it is organized around diverse and situated forms of musical legitimacy.

Gender offers another test of the consistency of access. Beliefs about instruments considered “appropriate” for girls or boys can condition choices, expectations, and musical self-perception from school age (Concina and Gesuato, 2025). In higher education, the positions of prestige and periphery described by Borgström Källén and Ferm Almqvist (2025) show how mechanisms of legitimacy continue into later educational trajectories. Multimodal access and situated representation are therefore evaluated by asking which ways of sounding, moving, leading, and learning have been recognized as valid.

### Recognized participation and voice

For this second mechanism, the comparative reading of literature on special educational needs, ensemble relationships, university music clubs, queer and gender studies, and assessment highlighted two related categories: recognized contribution and influence within shared practice. Recognized contribution refers to whether students’ participation is valued by teachers, peers, and the musical group. Influence refers to whether students can affect decisions, roles, feedback processes, or the development of the musical activity. These categories informed the mechanism of recognized participation and voice.

Recognized participation distinguishes presence from contribution with value. The review by Mommo et al. (2025) shows that the participation of students with special educational needs improves when student-centered pedagogies, peer interaction, co-teaching, and varied methods are used. This evidence supports a demanding definition of inclusion: students do not simply occupy a place in the activity; their forms of response, exploration, and learning receive sufficient support to become part of the common practice.

Voice is used here in a restricted analytical sense. It does not refer to every form of expression, identity recognition, or belonging, but to the possibility that students’ contributions can influence the shared musical practice. In this sense, voice includes taking initiative, influencing musical decisions, proposing variations, occupying diverse roles, receiving feedback that allows further participation, and being heard in the organization of the musical space. Ryan et al. (2025) showed that relationships within ensembles are associated with belonging, leadership, atmosphere, and identity. Guo et al. (2026) linked participation, perceived recognition, and expressive safety with belonging, identity, and agency in university clubs. These studies help specify the relationship between voice, recognition, and the possibility of influencing shared practice.

Recent literature on queer and gender studies in music education deepens this notion of voice. Voice is not limited to musical intervention or choosing among already available options; it also refers to the possibility that certain identities, trajectories, and experiences are named, heard, and recognized within the musical environment. Beauregard’s (2026) work on bisexual music educators and the “silent B” in LGBTQ+ discourse is useful for considering forms of silencing that may persist even within apparently inclusive frameworks. More broadly, the volume edited by McBride and Sears (2026) shows that questions about gender and queer studies in music education affect language, visibility, trust, representation, and institutional conditions of participation.

Assessment is discussed within this mechanism because it affects whether students’ contributions are valued and whether they can continue to participate with confidence and influence. Recognized participation therefore also requires reviewing assessment. Parker and MacLeod (2026) showed that nonmusical factors, such as race and interactions with gender and instrument, can influence performance evaluations. Zhu and Li (2026) argued that the teaching of music theory requires gender-responsive designs because confidence, participation, and self-regulation mediate access to specific cognitive tasks. These works warn that voice can be limited when achievement criteria appear neutral while reproducing biases related to repertoire, instrument, confidence, embodiment, or authority.

### Expressive safety, belonging, and situated wellbeing

This third mechanism was developed from literature on wellbeing, self-esteem, belonging, ensemble relationships, and complementary therapeutic approaches. In the synthesis, these sources converged around the relational regulation of expressive exposure, error, judgment, identity affirmation, and belonging. Unlike the previous mechanism, which focuses on whether students’ contributions can influence the shared musical practice, this mechanism focuses on the conditions that make participation emotionally and relationally sustainable.

Expressive safety refers to the possibility of trying, making mistakes, modifying a response, asking questions, remaining temporarily silent, or participating gradually without error becoming humiliating exposure. In musical contexts, this condition carries particular weight because many tasks involve bodily visibility, vocal production, coordination with others, improvisation, public performance, or aesthetic judgment (Goopy and MacArthur, 2025; Jiang and Tong, 2025; Ryan et al., 2025). Expressive safety is not equivalent to a generally pleasant climate; it is observed in the organization of turns, waiting times, feedback, leadership distribution, peer listening, and graduated opportunities for achievement.

The need for expressive safety is also supported by the existence of negative or exclusionary musical experiences. Musical environments may involve performance anxiety, fear of error, competitive pressure, elitist norms, audition practices, racism, ableism, sensory overload, cultural tokenism, unequal access to leadership, or gradual withdrawal from participation. These experiences reinforce the caution that music is not inherently inclusive. Its inclusive value depends on how exposure, error, repertoire, assessment, access to roles, and recognition are organized.

Wellbeing is treated here cautiously as a situated psychoeducational dimension associated with the relational quality of musical participation, rather than as a direct outcome demonstrated by expressive safety alone. Jiang and Tong (2025) found a positive relationship between music education and self-esteem, while noting limitations associated with the predominance of cross-sectional designs and uneven methodological quality. Goopy and MacArthur (2025) reviewed the relationship between music learning and wellbeing in school-aged students, describing individual, social, and educational outcomes alongside conceptual and methodological diversity. The framework treats these contributions as indicators of environmental quality: belonging, confidence, musical self-esteem, self-regulation, social connection, and perceived competence.

Accordingly, the relationship between expressive safety and wellbeing is presented as a conceptual proposition derived from the synthesis, not as a causal relationship established by the reviewed studies.

The literature on musical belonging helps refine this argument. Ryan et al. (2025) showed that belonging in ensembles is related to interpersonal bonds, leadership, atmosphere, identity, and wellbeing. These findings suggest that expressive safety does not depend only on students’ individual dispositions, but on how the environment regulates exposure, recognition, and the possibility of contributing without participation being evaluated solely through error. In this sense, a musical practice can support wellbeing when it reduces disproportionate exposure to judgment and broadens the legitimate ways of showing understanding, initiative, and musical presence.

Complementary therapeutic literature helps specify relational dimensions of expressive safety that educational research does not always develop explicitly. Baines et al. (2019) argued for the need to include queer theory in music therapy training in order to question hetero- and cisnormative assumptions. Biedka (2022) analyzed therapeutic relationships with people with marginalized gender identities and sexual orientations, emphasizing the importance of alliance, recognition, and professional sensitivity. Gross (2023) proposed shifting music therapy away from the medical model of disability toward an intersectional understanding. Mulley et al. (2026) showed that LGBTQIA+ participants and music therapists regard non-discrimination, recognition, and specific knowledge as fundamental. Ribeiro et al. (2025) called for intersectional, antiracist, feminist, and LGBTQIA+-affirmative perspectives in music therapy. Taken together, these works orient the educational analysis toward practices that reduce disproportionate exposure to judgment, broaden identity recognition, and reduce the persistent assignment of some students to secondary or peripheral roles.

These therapeutic studies are not used as direct evidence for school-level educational effects. Rather, they are used heuristically to refine relational principles that are also relevant to educational environments: recognition, affirming language, awareness of power asymmetries, identity safety, and protection from disproportionate exposure to judgment. This transfer should therefore be understood as conceptual and limited, requiring empirical examination in educational contexts.

The three mechanisms should be understood as interdependent rather than sequential. Multimodal access and situated representation concern the conditions through which students are allowed to enter musical practice and encounter forms of musicality that can be recognized as legitimate. Recognized participation and voice concern whether this access becomes contribution, initiative, and influence within the shared practice. Expressive safety, belonging, and situated wellbeing concern the relational conditions that regulate exposure, error, evaluation, and recognition. In this sense, access without recognition may produce presence without meaningful participation; recognition without expressive safety may expose students to judgment without adequate relational protection; and expressive safety without access and voice may generate a positive atmosphere without redistributing participation.

Teacher facilitation and institutional responsibility are treated here as transversal enabling conditions rather than as a fourth mechanism. They do not describe a separate dimension of student participation; rather, they mediate the implementation of all three mechanisms. Teachers and institutions shape which modes of access are available, which contributions are recognized, how evaluation is organized, how expressive risk is regulated, and how inequalities linked to gender, disability, culture, race, class, or previous musical experience are addressed.

### Teacher facilitation and institutional responsibility

During the coding and comparative synthesis, teacher facilitation and institutional responsibility appeared across the categories associated with all three mechanisms: access design, repertoire selection, recognition of contributions, assessment, role distribution, expressive exposure, support, and institutional culture. They were therefore not treated as a fourth mechanism because they do not describe a separate dimension of student participation. Rather, they refer to the professional and institutional mediation through which multimodal access, recognized participation, and expressive safety are made possible or restricted.

The three mechanisms depend on professional and institutional mediation. The review by Mommo et al. (2025) identifies lack of training and resources as persistent barriers to inclusion in music lessons. Tomé-Fernández and Bravo-Yebra (2025) found that Spanish teachers perceive intercultural competence positively, but encounter obstacles related to training, curricular structure, and the availability of advice. Harkopf and Bylica (2023) showed that teacher preparation oriented toward social justice requires connections between theory and contextual application, because novice teachers face accountability pressures and tensions between critical practices and institutional demands.

Institutional responsibility is necessary because biases do not operate only through individual decisions. Borgström Källén and Ferm Almqvist (2025) describe how gender hierarchies persist in higher music education through symbolic positions, habits, and distributions of authority. Niknampour (2026) shows that recent changes in institutional diversity, equity, and inclusion policies in higher music education generate differentiated emotional and professional responses, especially among gender-marginalized students.

Structural and material barriers also shape musical participation. Access to instruments, private tuition, transport, school funding, curricular time, specialist teacher availability, adaptive technologies, physical accessibility, audition procedures, and opportunities to enter prestigious ensembles or leadership roles may determine who is able to enter, remain, and progress within musical environments. Equity therefore depends not only on the relational and pedagogical quality of musical activities, but also on the material and institutional conditions that make sustained participation possible.

On the basis of this synthesis, the pedagogical implications of teacher facilitation can be described as designing before intervening: contextualized repertoires, multimodal pathways, graduated supports, explicit assessment, rotating roles, careful feedback, and spaces for reviewing biases related to gender, culture, ability, or social background. These elements are not presented as findings from a single body of evidence, but as implications derived from the conceptual integration of the reviewed literature. This mediation is not a fourth mechanism; it is the professional and institutional condition that allows the three previous mechanisms to operate in concrete practices.

Although some pedagogical indicators are related across mechanisms, they are assigned in Table 2 according to their primary analytical function. The first mechanism concerns entry into musical practice and the legitimacy of repertoires, bodies, cultures, and modes of response. The second concerns whether participation becomes recognized contribution and influence within the shared practice. The third concerns the relational conditions that make participation emotionally and expressively sustainable. This distinction allows the mechanisms to remain analytically differentiated while acknowledging their interdependence.

**Table 2:** Mechanisms of preventive equity in educational musical environments.

Mechanism	Analytical question	Bibliographic basis	Pedagogical indicators
Multimodal pedagogical access and situated representation	Which pathways into musical learning are recognized as legitimate, and which repertoires/bodies/cultures occupy the center?	Eccles et al. (2021), Del Barrio and Arús (2024), Juntunen and Sutela (2023), Mommo et al. (2025), Campbell (2020), Crawford (2020), Tomé-Fernández and Bravo-Yebra (2025), Concina and Gesuato (2025), and Borgström Källén and Ferm Almqvist (2025).	Auditory, bodily, vocal, instrumental, visual, and creative pathways; contextualized repertoires; graduated supports; review of cultural and gender stereotypes.
Recognized participation and voice	Does presence become contribution with value and capacity to influence the practice?	Mommo et al. (2025), Ryan et al. (2025), Guo et al. (2026), Parker and MacLeod (2026), Zhu and Li (2026), Beauregard (2026), and McBride and Sears (2026).	Distribution of turns and initiative; diverse roles; formative and non-disqualifying feedback; transparent assessment; opportunities to propose, lead, or modify responses.
Expressive safety, belonging, and situated wellbeing	Does the environment allow learning without disproportionate exposure to error and generate experiences of competence, connection, and recognition?	Goopy and MacArthur (2025), Jiang and Tong (2025), Ryan et al. (2025), Baines et al. (2019), Biedka (2022), Gross (2023), Mulley et al. (2026), and Ribeiro et al. (2025).	Climate of trust; pedagogical management of error; identity recognition; inclusive language; belonging; musical self-esteem; self-regulation; social connection.

Table 2 summarizes the three mechanisms as analytical propositions derived from the integrative synthesis. The table does not present a validated measurement model; rather, it identifies conceptual dimensions and pedagogical indicators that can guide future empirical examination.

These mechanisms are intended to function as analytical criteria. Their empirical distinctiveness, internal coherence, and applicability across educational domains and student populations require further study.

## Discussion

This article shifts the analytical focus from the effects attributed to music education toward the conditions that regulate its inclusive value. The reviewed literature has documented associations among musical participation, wellbeing, self-esteem, belonging, social–emotional learning, and inclusion (Goopy and MacArthur, 2025; Jiang and Tong, 2025; Mommo et al., 2025; Ryan et al., 2025; Váradi, 2022). At the same time, these studies come from heterogeneous designs, populations, and conceptual frameworks, which limits direct comparability and makes it difficult to identify which pedagogical and relational conditions sustain those outcomes. From this perspective, the proposed framework addresses a less examined question: under what conditions can musical practices transform formal access into meaningful, recognized, and equitable participation?

A first consequence of this framing is a revised understanding of access. Much educational literature has shown that music can broaden learning pathways through the body, rhythm, listening, movement, cooperation, and expression (Del Barrio and Arús, 2024; Eccles et al., 2021; Juntunen and Sutela, 2023; Váradi, 2022). The discussion of inclusion, however, cannot be resolved solely in terms of methodological variety. Multimodality acquires equitable value when it reorganizes the criteria through which a musical contribution is recognized as valid. This distinction makes it possible to differentiate musically varied activities from equity-oriented environments: the former diversify resources; the latter redistribute possibilities for access, expression, and recognition.

Cultural and identity representation introduces a second dimension of the problem. Studies on interculturality, gender, and assessment show that repertoires, instruments, roles, and performance criteria are not neutral (Borgström Källén and Ferm Almqvist, 2025; Campbell, 2020; Concina and Gesuato, 2025; Crawford, 2020; Parker and MacLeod, 2026; Tomé-Fernández and Bravo-Yebra, 2025; Zhu and Li, 2026). This convergence supports a central idea of the framework: musical inclusion requires reviewing which forms of musicality occupy central positions and which remain peripheral. Situated representation, therefore, is not exhausted by the presence of cultural or identity diversity; it requires analyzing how that diversity participates, is heard, is assessed, and is recognized within the practice.

Wellbeing raises a third conceptual consequence. Reviews on self-esteem, wellbeing, and belonging show positive relationships between musical experience and psychoeducational dimensions, together with considerable conceptual and methodological diversity (Goopy and MacArthur, 2025; Jiang and Tong, 2025; Ryan et al., 2025). This synthesis avoids treating wellbeing as a general outcome of musical participation and places it within a concrete relational configuration. In musical contexts, wellbeing is linked to how bodily or vocal exposure, error, peer assessment, initiative, leadership, and recognition are managed. This reading connects wellbeing and equity: a musical practice may be positive for some students while producing withdrawal or peripheral participation for others when these conditions are not adequately regulated.

The notion of voice articulates these dimensions. Literature on belonging, queer studies, gender, and music therapy shows that participation does not necessarily mean having an influence on shared practice (Baines et al., 2019; Beauregard, 2026; Biedka, 2022; Gross, 2023; Guo et al., 2026; McBride and Sears, 2026; Mulley et al., 2026; Ribeiro et al., 2025; Ryan et al., 2025).

Within this framework, voice is not reduced to individual expression or to choosing among pre-established options. It refers to the possibility that students’ contributions modify the practice, are recognized by the group, and are not disproportionately constrained by hierarchies of gender, culture, ability, instrument, confidence, or authority. This formulation expands the notion of participation by locating it at the intersection of recognition, agency, and expressive safety.

The concept of preventive equity synthesizes this contribution. Instead of conceiving inclusion as a response after a barrier has already been identified, the framework proposes analyzing in advance the conditions that may produce low participation, silencing, loss of confidence, or withdrawal. This reading is especially pertinent in music education, where many forms of exclusion do not necessarily appear as explicit expulsion; they take more gradual forms, such as repeated assignment to secondary roles, less exposure to leadership positions, biased assessment, repertoires with little meaning for students, or forms of participation that are tolerated but not valued. Preventive equity makes it possible to name these processes before they consolidate into unequal trajectories.

The framework also delimits the scope of transfer across fields. School-based evidence provides the most direct basis for analyzing pedagogical support, emergent literacy, music-movement, wellbeing, and inclusion of students with special educational needs. Literature from higher education, ensembles, and music clubs helps specify processes of belonging, leadership, agency, assessment, and institutional culture. Queer, trans, and intersectional music therapy contributes to conceptualizing relational safety, language, recognition, and power. The contribution consists of identifying comparable mechanisms without homogenizing contexts whose empirical manifestation must be examined according to educational level, population, and institutional structure.

The framework should therefore be understood as a three-mechanism organization composed of interrelated dimensions. Multimodal access and situated representation are grouped together because they concern the initial conditions of legitimate entry into musical practice: how students can participate and which repertoires, bodies, cultures, and forms of musicality are recognized as valid. Recognized participation and voice are grouped together because they concern whether presence becomes contribution with value and influence within the shared practice. Expressive safety, belonging, and situated wellbeing are grouped together because they concern the relational conditions that make participation emotionally and expressively sustainable. These mechanisms interact continuously: access may remain formal without recognition, recognition may become fragile without expressive safety, and a safe atmosphere may remain insufficient if it does not redistribute access and voice. Teacher facilitation and institutional responsibility operate across the three mechanisms by shaping access design, recognition practices, assessment, expressive exposure, support, and institutional culture.

Taken together, the discussion positions musical inclusion as a problem of pedagogical, relational, and institutional design. The three mechanisms allow the analysis of how legitimacy, initiative, recognition, and protection from disproportionate exposure to judgment are distributed. Thus, the framework avoids adding another list of benefits associated with music and instead offers a tool for evaluating the conditions under which those benefits become plausible, situated, and equitably distributed.

### Limitations and future research

This integrative narrative review has four limitations. First, because the review is integrative and narrative rather than systematic, the selection, interpretation, and synthesis of heterogeneous literature involved a greater degree of author judgment. This may limit reproducibility and increase the possibility of selection or interpretive bias. The purpose of the review was conceptual and organizational rather than to estimate aggregate effects. Accordingly, the framework should be understood as an interpretative synthesis of the reviewed literature rather than as a validated empirical model. Second, the available literature is uneven across educational domains. There are robust reviews on music-movement pedagogy, emergent literacy, wellbeing, and special educational needs, but fewer comparable studies examining how these dimensions interact in diverse music classrooms. Third, part of the literature on belonging, agency, identity, and expressive safety comes from higher education, ensembles, community music contexts, or music therapy. These sources were used as complementary evidence to refine relational and identity-related dimensions, not as a direct basis for school-level generalizations. Fourth, the concepts of wellbeing, inclusion, participation, belonging, and voice are operationalized differently across studies, which limits direct comparability.

Future research should examine how the three mechanisms operate in concrete educational settings. Longitudinal studies, mixed-methods designs, classroom-based research, and intersectional analyses are needed to observe how gender, disability, cultural background, social class, racialization, neurodivergence, and previous musical experience shape participation in musical environments. Further research should also test the framework itself: whether the three mechanisms are empirically distinguishable, whether the dimensions grouped within each mechanism are internally coherent, and whether the same organization remains meaningful across different educational domains, musical practices, and student populations. Developing pedagogical observation instruments could help evaluate multimodal access, recognized participation, and expressive safety in school, community, and training-based musical contexts.

## Conclusion

Music may support equity in educational contexts when it is designed as a practice of broad participation, recognition, and expressive safety. Recent evidence links music education with emergent literacy, movement, self-regulation, self-esteem, belonging, social–emotional learning, and inclusion, but these relationships should be understood in relation to the pedagogical and relational quality of the environment. Music in the abstract does not guarantee wellbeing, inclusion, or equity; conditions of access, support, assessment, repertoire, language, and recognition shape whether a shared musical experience becomes educationally inclusive.

The proposed framework organizes this argument around three conceptual mechanisms: multimodal pedagogical access and situated representation; recognized participation and voice; and expressive safety, belonging, and situated wellbeing. Its usefulness lies in connecting school-based, expanded educational, and therapeutic literature without confusing their scope. School-based studies provide the most direct basis for educational claims; expanded educational literature helps specify participation, leadership, agency, assessment, and institutional culture; and therapeutic literature contributes conceptual tools for understanding recognition, language, relational safety, and power.

This formulation offers a preventive reading of musical equity. Instead of waiting for low participation, withdrawal, silencing, or loss of confidence to appear, educational design can anticipate barriers and redistribute opportunities from the outset. The contribution of the article is to specify the conditions under which the benefits attributed to music may become plausible, situated, and pedagogically defensible. As a conceptual proposal derived from an interpretive synthesis of heterogeneous literature, the framework requires empirical examination in future studies to assess whether the three mechanisms are distinguishable, internally coherent, and transferable across different educational contexts and student populations.
